# Beyond the episodic–semantic continuum: the multidimensional model of mental representations

**DOI:** 10.1098/rstb.2023.0408

**Published:** 2024-09-16

**Authors:** Donna Rose Addis, Karl K. Szpunar

**Affiliations:** ^1^ Rotman Research Institute, Baycrest Academy for Research and Education, Toronto, ON M6A 2E1, Canada; ^2^ Department of Psychology, University of Toronto, Toronto, ON M5S 3G3, Canada; ^3^ School of Psychology, The University of Auckland, Auckland 1010, New Zealand; ^4^ Department of Psychology, Toronto Metropolitan University, Toronto, ON M5B 2K3, Canada

**Keywords:** autobiographical memory, episodic memory, future thinking, semantic memory, simulation, imagination

## Abstract

Tulving’s concept of mental time travel (MTT), and the related distinction of episodic and semantic memory, have been highly influential contributions to memory research, resulting in a wealth of findings and a deeper understanding of the neurocognitive correlates of memory and future thinking. Many models have conceptualized episodic and semantic representations as existing on a continuum that can help to account for various hybrid forms. Nevertheless, in most theories, MTT remains distinctly associated with episodic representations. In this article, we review existing models of memory and future thinking, and critically evaluate whether episodic representations are distinct from other types of explicit representations, including whether MTT as a neurocognitive capacity is uniquely episodic. We conclude by proposing a new framework, the Multidimensional Model of Mental Representations (MMMR), which can parsimoniously account for the range of past, present and future representations the human mind is capable of creating.

This article is part of the theme issue ‘Elements of episodic memory: lessons from 40 years of research’.

## 1. Introduction

The term ‘mental time travel’ (MTT) refers to the complex ability to mentally project oneself beyond the present and to (p)re-live experiences at another time in one’s personal chronology. That is, one can recollect a unique event one experienced in the past or imagine an event that one may experience in the future. Forty years since Tulving [1, p. 124] introduced the concept of MTT in his influential book, *Elements of episodic memory*, MTT is now almost synonymous with *episodic* forms of memory and future thinking, sharing multiple defining characteristics, including the mental displacement of the self to a specific moment beyond the present via the (re-)construction of one’s experience at that time. Because these qualities are fundamental to both episodicity and MTT, it has been difficult for scholars to conceptualize MTT as anything other than a primarily episodic phenomenon. Recent years have seen increasing consideration of the role that semantic memory plays in supporting constructing episodic representations, as well as conceptualizations of episodic and semantic memory as existing at opposite ends of a continuum interposed with various ‘hybrid’ forms of representations. Here, we critically review existing models of memory and future thinking, and in particular consider whether MTT is uniquely associated with episodic representations. We end by presenting a new multidimensional framework that broadens the notion of MTT to include non-episodic representations.^1^

## 2. Models of memory and future thinking

### (a) Tulving’s episodic–semantic distinction

Dichotomies are useful heuristics when exploring mental processes. Tulving [2] originally proposed the episodic–semantic distinction as a ‘pretheoretical’ first step in organizing a young field’s observations about memory, noting that it was not ‘some new theory of memory’ [2, p. 384]. Despite these caveats, the interpretation of Tulving’s distinction as separate, contrastive and cleanly delineated forms of memory has become a fixture of modern memory science [3,4].

Contemporary definitions of episodic and semantic memory draw heavily on Tulving’s [1,5,6] writings, distinguishing the two along dimensions such as (i) representational format (experiences versus facts); (ii) modality of constituent details (primarily perceptual versus conceptual); (iii) generalization across people (idiosyncratic/autobiographical versus culturally shared); (iv) generalization over time/space (unique versus repeated); (v) contextual reinstatement (contextual versus acontextual); and (vi) self-reference/autonoesis (self versus other, autonoetic versus (a)noetic). Taken together, episodic and semantic memory appear very different, as what Andonovski [7] refers to as ‘idealized’ systems. Episodic memories are representations of first-hand idiosyncratic experiences of the self, including one’s context during a specific event in subjective time, represented perceptually and organized primarily in terms of temporal relations. In contrast, semantic memories are culturally shared and generic knowledge, such as facts, concepts and schemas, decontextualized and without self-reference, represented symbolically and organized in terms of conceptual relations. These characteristics have been extrapolated to future thinking, where simulations of specific personal events are constructed using episodic details [8,9], while semantic future thinking refers to knowledge about general states, particularly the collective and non-personal [10–12].^2^

This dichotomy is manifest in half a century of theoretical and empirical developments. Nevertheless, it is important to take a step back to consider that Tulving’s episodic–semantic distinction was not nearly as sharp as more recently supposed. Tulving acknowledged that episodic and semantic memory overlapped considerably in representational format, relational organization and content; both are propositional and representable, organized as objects and relations that can be attended to internally via introspection and described (i.e. declarative [1,2,13]). Although Tulving emphasized the relative importance of spatiotemporal relations in episodic memory, he also argued that items in semantic memory are not only organized conceptually but also spatiotemporally, enabling inference. Similarly, while conceptual content is relatively more important for semantic memory, episodic memories also contain the conceptual understanding of experience, a point we return to below. In essence, Tulving left open the possibility that what he termed ‘episodic’ and ‘semantic’ memories could, in fact, be different expressions of the same memory system. Nevertheless, this dichotomy has become entrenched as a sharp division, including in Tulving’s own later work [14]. The distinctions between episodic and semantic memory have been magnified by ‘drift’ over time in the questions asked, methods used, and the evidence acquired [15]. Recently, there has been renewed interest in the interactivity between episodic and semantic memory [15,16], albeit still preserving the existence of two systems. Duff *et al*. [15, p. 12] have called for a deeper assessment of whether the ‘psychological and anatomical reality’ indicates that episodic and semantic memory can in fact be differentiated. We apply these considerations in our review of existing models of memory and future thinking, and reconsider whether the capacity for MTT is uniquely episodic.

### (b) Episodic–semantic continuum models

**Figure 1.**
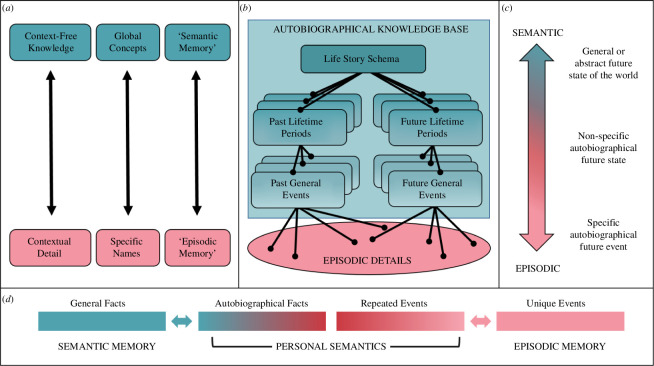
Diagrams of continuum models. (*a*) Levels of Processing [17,18]. (*b*) Self-Memory System [20,21]. (*c*) Taxonomy of Prospective Thinking [22]. (*d*) Episodic–semantic continuum including personal semantics [23].

A number of theories argue that, although distinct, episodic and semantic systems are highly interactive and overlapping, and better conceptualized as opposite ends of a continuum. Some of these theories were thought out in relation to memory, some have been adapted to encompass both memory and future thinking, and others have focused more specifically on future-oriented cognition. In all cases, however, these models envision episodic and semantic cognition as existing on a continuum. Despite a recent groundswell, this idea is apparent in earlier influential theories of memory. Craik & Lockhart ([17]; see also [18,19]) argued against the existence of separate memory ‘stores’ or systems that process different types of information with distinct attributes. Instead, their Levels of Processing framework proposed a continuous processing hierarchy ranging from ‘surface’-level processing of sensory-perceptual inputs to ‘deeper’ analysis of identity, meaning and implications. Importantly, Craik & Lockhart’s hierarchy can be applied to all types of information, including autobiographical events, whereby generalities (‘semantic’ autobiographical memory) are abstracted from multiple specific instances (‘episodic’ autobiographical memory) (figure 1*a*). By this view, Craik [18] argues that specific semantics (e.g. names) are just as vulnerable to loss as specific events, and abstract event representations are just as easily accessed as higher-level concepts. This is not to say that specific ‘surface’ attributes or singular instances cannot be processed deeply; specific information can be related to other information on the same or higher levels, affording it meaning and significance.

Other models are less continuous in nature, proposing different memory representations that vary in certain dimensions. Conway’s influential Self-Memory System model conceives of an autobiographical knowledge base organized hierarchically according to temporal specificity and abstractness (figure 1*b*) [20,21]. As a partonomic hierarchy, information is nested between levels. Individual sensory-perceptual records of experience (‘event-specific knowledge’ or ESK) are the most specific; the generalities of ESK shared among multiple events are organized into general event representations. In turn, multiple general events are synthesized into higher-order lifetime periods, which are woven together to form the life story. Notably, more abstracted forms of autobiographical knowledge are proposed to integrate across temporally separated events that are linked thematically or causally, often by overarching goals [24].

Because the Self-Memory System spans multiple levels of temporal specificity, researchers have implicitly or explicitly mapped this hierarchy to an episodic–semantic continuum where ESK is episodic and the most abstract representations (i.e. life stories) are also the most semanticized. Interestingly, Conway & Pleydell-Pearce [20, p. 272] stated that their distinctions between different types of autobiographical knowledge ‘do not fit well with concepts such as episodic and semantic memory’. They did, however, entertain the notion that general events and lifetime periods may be forms of semantic knowledge, and speculated that something akin to an episodic memory could be emergent from the integration of ESK with general events. Conway [25] added ‘episodic memory’ to the Self-Memory System at the level of ESK and separate from the ‘conceptual’ (or semantic) types of autobiographical knowledge (figure 1*b*). This demarcation enabled a direct mapping between the Self-Memory System and Tulving’s episodic–semantic distinction that is prominent in the autobiographical memory literature (e.g. [26,27]).

Renoult *et al*. [23] expand on the Self-Memory System model by explicitly mapping autobiographical memory to a continuum on which representations vary in abstractness and personal relevance, with non-personal ‘general’ semantics opposing episodic memory (figure 1*d*). They argue that several ‘hybrid’ types of autobiographical representations, referred to collectively as ‘personal semantics’, overlap with episodic and semantic memory to differing degrees depending on the weighting of certain attributes. For instance, repeated (general) events are closer to the ‘episodic end’ of the continuum because, although lacking in temporal specificity, they possess spatial organization [28] and sensory-perceptual imagery, albeit to arguably lower degrees than episodic memories. Moving towards the semantic pole, memories become more abstract and less personal in nature. Autobiographically significant concepts are ostensibly semantic because they are culturally shared concepts with added autobiographical relevance afforded by a link to a specific personal experience. Autobiographical facts, such as personally relevant names, dates and addresses, are considered more abstract and ‘skeletal’ and are thus, on average, closer to general semantics. Nevertheless, autobiographical facts can vary in how ‘experience-near’ they are, with some facts tightly associated with specific events [29].

Szpunar *et al.* [22] proposed a similar continuum of prospective representations that vary in both abstractness and personal relevance (figure 1*c*). At one end are episodic representations, such as imagining an event involving the self at a specific point in the future. At the semantic end, representations are both abstract and non-personal, referring to future states of external entities (e.g. an organization) or the world more generally. Intermediate are temporally general yet autobiographical representations, such as general future events, future-oriented personal semantics, states and goals. Although Szpunar *et al.* argued that their episodic–semantic continuum ‘provides a useful framework by which to discriminate and develop connections among various forms of future thinking’ [22, p. 18420], they were also careful to note that their framework was designed as ‘an impetus toward delineating the multidimensional ways in which the various modes of future thinking interact with one another’, anticipating that a ‘revised taxonomy’ would likely be needed [22, p. 18419].

## 3. Critiques of continuum models

Although conceptualizing memory and future thinking as existing along a continuum has intuitive appeal, some have questioned its usefulness. The very notion of a continuum supposes, first, that episodic and semantic representations are distinct, with ‘pure’ forms of each at opposite poles, and second, that such representations can be differentiated along a single axis (‘A continuum of what?’ [1, p. 67]). In practice, however, both suppositions are problematic. In order to operationalize and map continuums to behaviour, often one or more binary distinctions (or ‘qualitative distinctions’ [23]) are used to create discrete types of representations. If a representation is not strictly episodic because it is not temporally specific, it is usually considered more-or-less semantic. Notably, however, the same principle is not applied at the other end of the continuum: if a representation is not strictly semantic, it is not usually considered more-or-less episodic, implying some special status for episodic representations (but see [7,30,31]). Moreover, the semantic pole is demarcated from the rest of the continuum by its non-personal or ‘general’ nature^3^ (e.g. [22,23]). Finally, while specific event representations (and to a lesser extent, repeated events) comprise predominantly perceptual and spatiotemporal detail, semantic representations are more abstracted and symbolic. Thus, continuum models typically conflate temporal specificity, content and self-reference on the same ‘episodic–semantic’ axis. As a result, many ecologically valid representations cannot be easily accommodated within these frameworks.

### (a) Temporal specificity

Episodic representations are distinguished theoretically from other forms because they possess temporal specificity; any temporally ‘general’ representation is usually considered to be abstract and non-episodic. However, the operationalization of ‘temporal specificity’ is itself problematic. One conceptualization of temporal specificity relates to regularity; semantic memory represents highly regularized patterns of experience, while episodic memory represents exceptions [32]. In this way, episodic memory represents unique ‘moments’ in time [1] rather than longer spans of time over which regularities emerge (e.g. lifetime periods). Consequently, in the autobiographical memory (and episodic future thinking) traditions, a temporally specific representation reflects experience on the order of ‘minutes, hours or a day’ [33, p. 801].

However, the segmentation of ongoing experience is somewhat arbitrary and may not reflect the reality of experience. Consider, for instance, a participant’s contextually unique and richly experiential description of childbirth which, because it extends past the 24 h mark, is considered non-episodic [34]. On the other hand, single episodes can be very short—seconds to minutes—as bounded by context changes (e.g. walking into a new room [35]). In this way, a day-long episode could be considered a string of event segments, similar to Tulving’s [1] idea that mini-events can be nested into longer episodes (e.g. words on a list). With this supposition, Conway & Pleydell-Pearce’s [20] classification of ‘extended events’ as non-episodic becomes problematic, given that they are also strings of shorter ‘episodic’ representations, albeit over a longer timespan (e.g. various day trips during a week-long vacation). Perhaps the ‘non-episodic’ status was meant to account for whatever links these events over a timespan longer than a day. Moreover, while lifetime periods reflect commonalities abstracted across events within a given time period, such as people, locations and activities, they also integrate these stable aspects of experience with life changes of varying scales that are often temporally specific (e.g. the death of a loved one; [36]). Overall, it remains unclear whether events can be cleanly separated into episodic and non-episodic by their temporal width, and whether MTT is only possible for shorter experiences. We argue that there are no sound theoretical reasons to do so.

Temporal specificity is also argued to be an attribute of the episodic representation itself—a ‘time-stamp’ or temporal ‘tag’ that later serves to localize the event within subjective time [37,38]. However, there is compelling evidence to suggest that temporal information—such as temporal locations (‘last spring’, ‘midday’, past versus future)—is not encoded as part of episodic representations [31,39–42]. Instead, temporal information tends to be rapidly inferred online from the contents of the event representation itself by a triangulation of the elements of an event and links between them (i.e. people, location, weather, activities), associated facts (e.g. I moved in January) and rich knowledge of temporal patterns (e.g. snow falls in winter). Importantly, this accounts for phenomena such as recalling fine temporal information (e.g. time of day) while simultaneously failing to retrieve larger-scale temporal details of events (e.g. year). As Friedman argues [31, p. 323], the metaphor of mentally travelling through time ‘unintentionally impl[ies] a unity and continuity of time that is quite at odds with the fragmentary, manifold way humans experience it’. Furthermore, the online inference of temporal location provides a common mechanism that applies equally to multiple types of non-episodic representations (e.g. lifetime periods, general events, and facts about temporally specific events), as well as simulations of events that are yet to occur [41]. Taken together, there is no compelling reason to believe that specific episodic events represent time any differently from other forms of memory or future thinking. Instead, the temporal ‘re-experiencing’ that we typically associate with episodic representations is a function of inferences made based on constituent elements and their inter-relations; as such, this mechanism could equally be at play during the retrieval of other complex multidimensional representations (for related discussion, see [43]).

### (b) Content

Episodic representations are also conceptualized as comprising rich contextual detail that is ‘experience-near’ and thus presumed to be primarily sensory-perceptual (particularly visuospatial). It is notable, then, that there is general agreement that episodic memories and future events are not limited to ‘episodic’ content [26,44,45], and routinely include autobiographical facts, self-knowledge, temporally general events and general semantic knowledge [46,47]. The inclusion of semantic content may reflect, at least in part, the contextualization of specific events within broader knowledge structures during retrieval or simulation [21], possibly as a way to scaffold event (re-)construction [48,49]. On the whole, however, episodic representations are thought to comprise more episodic than semantic content (see [26], for discussion of factors influencing relative proportions of episodic and semantic content). The relative amount of episodic content denotes ‘episodic specificity’, but only if the representation itself meets criteria for ‘episodic specificity’ (i.e. temporal specificity and self-relevance; [44]).^4^ These observations suggest that episodic representations can also be considered hybrids, raising important questions of what constitutes a ‘hybrid’ representation, and whether episodic hybrids differ qualitatively from personal semantic ones [23,29].

Interestingly, many neuroimaging studies report similar default mode network (DMN) activation patterns for semantic and episodic tasks (e.g. [53,54]). A prominent explanation appeals to the hybrid nature of episodic memories. The retrieval of episodic memory activates DMN regions mediating both episodic and semantic memory because episodic memories comprise both episodic and semantic content. A similar argument has been advanced to explain the reliance of personal semantic hybrids on both ‘semantic’ and ‘episodic’ DMN regions (e.g. [29]). Recent theories of episodic memory and future thinking (e.g. [30,55–58]) argue that semantic and schematic content is mediated by medial prefrontal and anterolateral temporal cortices, whereas episode-specific perceptual content is mediated by medial parietal and temporal cortices. A similar anterior-to-posterior gradient is argued to exist in the hippocampus, with conceptual-to-perceptual detail processed along its long axis. Thus, the hippocampus is thought to serve as the interface between the episodic and semantic systems to produce hybrid episodic representations.

It is noteworthy that these theories still retain the idea of distinct episodic and semantic neurocognitive systems, by conceiving of ‘interactions’ between the two. Contrary to the existence of episodic and semantic DMN sub-systems is evidence that the general semantic system resides across much of the DMN, including arguably ‘episodic’ regions such as angular gyrus, posterior cingulate, precuneus, parahippocampal and retrosplenial cortices. Indeed, Binder *et al*. [54, p. 2781] note that the ‘nearly complete overlap observed in functional imaging studies is striking’. The DMN is well positioned anatomically, at the confluence of multiple sensory systems, affording the capacity for abstracting across and combining multimodal inputs to construct conceptual representations. Thus, another explanation is that some, if not all, of the supposedly ‘episodic’ content composing event representations is actually conceptual (for similar arguments in the context of episodic future thinking, see [9]).

As noted above, Tulving [2,5] acknowledged that episodic memories contain our conceptual understanding of our experience, and, at retrieval, both episodic and semantic referents are likely co-activated and impossible to separate. Indeed, Binder *et al.* [54, p. 2781] assert that ‘autobiographical memories are necessarily composed of concepts and that there could be no retrieval of an autobiographical memory without retrieval of concepts’. More recently, Renoult *et al.* [16] have conceded that the activation of some DMN regions during episodic retrieval may reflect the reinstatement of conceptual processing during the original experience, albeit along with the ‘episode-specific’ sensory and spatial aspects of the episode. It is possible that the representational format of different elements of a representation depends on familiarity. For instance, even within a very specific event, highly familiar or schema-consistent entities (e.g. a significant other; the stove in our kitchen) may be represented conceptually [59–63].

### (c) Self-reference and autonoesis

According to Tulving [1,6,13], the most distinctive and defining feature of episodic memory is the awareness of the self situated in subjective time. Termed autonoetic consciousness, this capacity enables the present self to engage in MTT and (p)re-experience or (p)re-live its own past and future. Tulving [6, p. 5] argued that autonoetic awareness is a necessary condition for episodic memory; one ‘cannot remember without awareness’ . We note that self-reference and autonoesis often go hand in hand as the experience of subjective time is believed to occur in the context of one’s personal chronology. However, these are also dissociable constructs. For instance, self-reference, such as the extent to which a particular object, person or place holds relevance to oneself, can be considered outside of the context of autonoesis. Importantly, for our purposes, neither self-reference nor autonoesis is necessarily tied specifically to episodic content.

Episodic representations are considered highly referential as they represent idiosyncratic events experienced by the self first-hand. In contrast, semantic representations lack self-reference and are associated with noetic consciousness because they is culturally shared knowledge about the world. However, there is not a 1 : 1 mapping of self-reference and ‘episodicity’, with many non-episodic representations being self-referential (e.g. personal semantics, autobiographical knowledge). Furthermore, self-reference is not a simple binary distinction. It varies according to the centrality of a representation to one’s self as well as with psychological distance, both temporal and social (e.g. close versus distant others or collectives; [64,65]). Moreover, the degree of self-reference does not track linearly with episodicity; for example, despite being the least episodic, life stories are arguably the most self-referential representations we possess, capturing the essence of the self over subjective time [66].

Autonoesis is predicated on the involvement of the self, but to differentiate episodic representations from other self-referential forms of memory, it is also associated with the richness of episodic trace. Tulving’s logic was that recovering one’s first-hand experience (i.e. context) promotes ‘reliving’ and a certainty that this event was indeed experienced previously by the same self who is now remembering [13]. This is captured via phenomenological ratings of representational quality (e.g. vividness, mental imagery; [67]) or whether ‘something they experienced at the time’ of encoding is recollected (i.e. ‘remember’ versus ‘know’; [6,68]). As such, the concept of autonoesis has become entangled with the reinstatement of experiential content.

In contrast, Conway and colleagues [21,25] argue that autonoetic consciousness results from the interaction of episodic details with conceptual forms of autobiographical memory and the self. Thus, the strength of autonoesis is not a function of episodic detail but the embedding of an event in autobiographical knowledge. Klein [69, p. 386] argued for decoupling autonoetic consciousness from contextual content. He describes individuals with amnesia who have re-acquired their past experiences, the resulting memories being appropriately self-referential, spatiotemporally specific, and perceptual—but lacking in the ‘feeling of reacquaintance with the act of acquisition’. Similarly, healthy individuals engage in episodic retrieval without autonoesis (i.e. ‘know’ responses to stimuli on an episodic task; [68]). Taken together, self-reference and autonoesis are unlikely to be unique to, or scale with, the episodicity of representations or the content of said representations.

## 4. Multidimensional models of memory

The above-noted issues associated with situating past and future representations along an episodic–semantic continuum result in much confusion in the literature, especially the autobiographical memory and future-thinking literatures, about whether a representation—and the elements composing the representation—are episodic or semantic. An alternative approach that has not received as much attention in the field is to allow representations to vary along multiple dimensions ([70–72]; see also [23]). We believe this approach holds promise for alleviating limitations inherent to continuum models, such as the conflation of features across ‘distinct’ kinds of memory, and is well suited to both memory and future thinking.

With respect to dimensional models, two important considerations are which dimensions should be modelled and whether those dimensions should be represented categorically or continuously. We consider these issues in the context of providing a brief overview of two existing multidimensional models of memory representations. As will become apparent, the decision of what dimensions to model ultimately comes down to what aspects of memory the researcher is interested in delineating. We then proceed to present a novel dimensional model that we argue best allows us to tease apart dimensions of past and future representations that are often confounded in the context of the episodic–semantic distinction and that can also be extended to other related forms of internal mentation.

**Figure 2.**
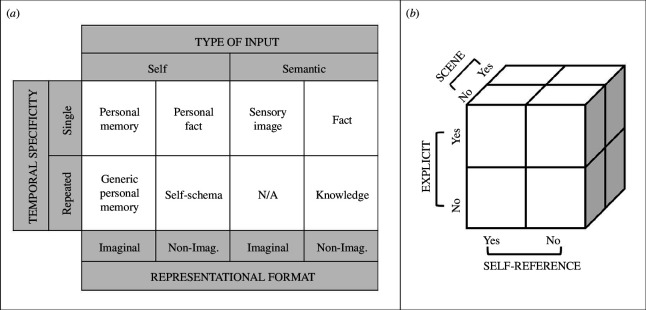
Multidimensional models of memory by (*a*) Brewer [70] and (*b*) Rubin [71]. Non-Imag. = non-imaginal; N/A = not applicable. (Brewer [70] argued that repeated semantic information with variation would not have imaginal properties.)

In an influential chapter on autobiographical memory, Brewer [70] aimed to situate an emerging field of autobiographical memory in the context of more traditional lab-based memory research. In this context, Brewer argued that organizing autobiographical and non-autobiographical memories as a function of their acquisition conditions (i.e. single versus repeated), representational format (i.e. imaginal versus non-imaginal), and type of information (i.e. self versus non-self, with the non-self category further fractionated into visual-spatial, visual-temporal and semantic) could help to situate the multitude of possible cognitions about the past in a common representational space (figure 2*a*). Under this configuration, episodic memory, or personal memory as Brewer referred to the category, is characterized as a single instance of personal imaginal content, whereas semantic memory or knowledge is characterized as repeated non-personal and non-imaginal content. Importantly, rather than assuming hybrid forms of representation such as autobiographical facts fall somewhere between episodic and semantic memory, they are more precisely defined as single instances of personal non-imaginal content.

More recently, Rubin [71,72] also proposed a dimensional model ofmemory with a focus on contrasting the effectiveness of dimensional approaches to classification relative to standard hierarchical models in the literature (e.g. [20,25]). Rubin based his model on Squire’s well known hierarchical model of memory [73], using the distinction between explicit and implicit representations as a central dimension for classifying memory. Rubin further focused on two additional dimensions that he considered to be well supported by the neuropsychological and neuroimaging literature, namely, whether a memory is event-based (i.e. tied to a spatial layout; [28,74]) and whether it is related to the self (figure 2*b*).

Unlike Brewer, Rubin stressed that the dimensions along which memory representations vary are continuous rather than dichotomous, an important point to which we return below. Nonetheless, Rubin reasoned that depicting his model as combinations of dichotomies would support ease of exposition and connection to the existing literature. Accordingly, Rubin’s three dimensions result in a set of eight memory categories. As with Brewer’s approach, Rubin’s model helps to situate episodic (i.e. explicit, event-based and self-relevant) and semantic (i.e. explicit but neither event-based or self-relevant) memories in a multidimensional space that allows a neater integration with other memory phenomena that are neither clearly episodic or semantic. For instance, under Rubin’s scheme autobiographical facts are labelled as explicit memories that are not event-based, but that are related to the self. Moreover, Rubin’s framework identifies combinations of dimensions that reveal gaps in our understandingof both the explicit representation and implicit influence of memory (e.g. implicit self-referential memory with or without scenes is characterized as the possible influence of memory in various anxiety disorders), demonstrating the generative potential of dimensional models. Such approaches are not without their limitations. Later, we highlight possible issues associated with using dimensional space to generate novel categories of memory phenomena.

As we alluded to above, there are many dimensions along which mental representations can vary in the context of such models. While this feature might be construed as a limitation of dimensional models of memory (e.g. see [7]), we argue that such inherent flexibility represents an advantage over continuum models such that dimensional models can situate various mental representations in a common space that does not privilege one representation over others or conflate dimensions across representations. This feature of dimensional models also allows the researcher to carve out dimensional space that may be particularly suitable to answer their question of interest, whether it is about the relation of autobiographical and lab-based representations [70], the implicit influence of memory and future thinking on behaviour [71,72], the delineation of non-depictive dimensions of representation that help individuals to distinguish between related but distinct modes of simulation [75], or many other possible questions that benefit from crossing multiple dimensions of representation rather than focusing on a single dimension. An important issue does arise, however, when the number of dimensions in a model begins to add up. For instance, when Rubin [71] considers the inclusion of emotion and certainty as additional dimensions of interest, the model quickly expands to 16 and 32 different categories of memory and it becomes increasingly tenuous as to whether each of the categories that fall out of such an approach reflects naturally occurring mentation in daily life. From this perspective, it is perhaps easy to understand why memory scientists tend to stick with simple, if incomplete, dichotomies. Indeed, Tulving [2] seemed to anticipate such issues when he referred to the episodic–semantic distinction as useful—but nevertheless a heuristic—for the field. However, we agree with Rubin [71,72] that the complexity of possible mental representations should not deter scientists from demarcating their constructs of interest as clearly as possible.

## 5. The Multidimensional Model of Mental Representations (MMMR)

**Figure 3.**
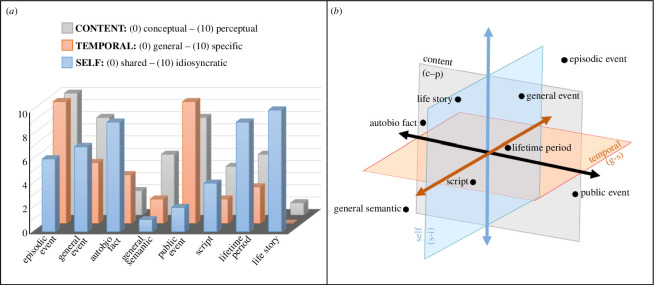
Visualizations of the Multidimensional Model of Mental Representations (MMMR), where eight distinct but related explicit mental representations are allowed to vary along three continuous dimensions: content (conceptual to perceptual, c*–*p); temporal specificity (general to specific, g*–*s); and self (shared to idiosyncratic, s–i). (*a*) The representations are characterized by scores on the three dimensions. (*b*) The representations are situated in three-dimensional space. autobio = autobiographical.

With these considerations in mind, we propose the Multidimensional Model of Mental Representations (MMMR), which, as we noted earlier, can be applied to explicit or declarative representations that emerge during internal mentation. This model aims to make clear the contributions of temporal specificity, content and self-reference—factors that tend to be conflated in the context of distinctions between episodic and semantic cognition—to mental representations by placing each dimension on a separate axis, such that mental representations can vary in the extent to which they are (i) temporally specific as opposed to general, (ii) perceptual as opposed to conceptual, and (iii) idiosyncratic as opposed to shared (figure 3). By disentangling these dimensions, our model creates a representational space that maximizes the classification of key concepts in the contemporary literature, in terms of both their differences and similarities, and provides a basis for making predictions about newly emerging areas of research. Critically, we treat these dimensional axes as continuous, which allows us to classify representations without the need for viewing any category as a natural kind. Rather, mental representations are coded as a particular constellation of features or components that when brought together result in cognitions that are associated with differing behavioural, phenomenological and neural correlates.

Our approach has a lot in common with that of Rubin [71,72], who, from a theoretical perspective, also suggested that dimensional models for organizing mental representations such as memory are best characterized as continua rather than dichotomies. One primary difference in our approach is the dimensions that we use to demarcate the representational landscape. By considering the role of explicit and implicit phenomena, Rubin opens the door to investigating the multitude of ways in which memory can impact thought and behaviour that goes beyond explicit mental representations of memory. Our focus is on maximizing the classification of explicit mental representations as they relate to everyday life. Although like Rubin we consider the implications of our model for organizing and making predictions about memory, we extend our model to future thinking. Moreover, our model is also capable of accounting for mental representations that are atemporal, such as those related to social cognition and narrative processing (e.g. [76]), and those that guide the perception of ongoing experience [57]. That is, we conceptualize mental representations as the reinstatement of neural processing related to specific or general, perceptual or conceptual, idiosyncratic or shared, elements of experience that can be flexibly integrated with one another to support a wide range of task performances that are not necessarily about memory or future thinking *per se*.

In figure 3, we demonstrate how permitting memory in particular to vary along the dimensions of temporal specificity, content and self-reference allows differentiation across eight distinct but related constructs that have each received extensive attention in the literature. As with the classification schemes proposed by Brewer [70] and Rubin [71,72], our approach gives not only ‘episodic’ (i.e. temporally specific, perceptual and personal) and ‘semantic’ (i.e. temporally general, conceptual and non-personal) cognitions a place in such representational space, but also hybrid forms such as autobiographical facts (i.e. temporally general, conceptual and personal). Moreover, it can situate other forms of non-personal collective representations often identified as semantic, including those that are temporally specific and perceptual, such as public events and simulations of vicarious and fictional events [77–80], as well as those that extend over considerable periods of time and are more conceptual (e.g. cognitions about World War II or the economic future of a nation [81,82]; for relevant reviews, see [83–85]).

Because our model does not elevate one kind of explicit representation over others it opens the door for new developments in the field. First, we hope that the arguments that we have elaborated on in this article serve as an impetus for the field to take stock of the enigmatic nature of the concept of an event or episode. There is good evidence that experience is segmented at many different levels of representation, ranging from moment-to-moment perception of ongoing experience (e.g. [86]) to transitional events that mark important boundaries across the lifespan (e.g. [64]), and neuroimaging work supports the idea that the brain does indeed represent even the same event at multiple levels of representation [87]. Moreover, while the field has gained many insights into the structure and functions of memory and future thinking in the context of studies that focus on specific events, it is becoming increasingly clear in the context of behavioural and neuroimaging studies of memory that general, schematic and conceptual factors play an important role in the processing and retention of ongoing experience (for more detailed discussion, see [57]). By allowing mental representations to vary in terms of their temporal specificity, content and self-reference, our model can accommodate this complex reality in a way that is not possible for continuum models that are based on single dichotomies that conflate multiple dimensions of representation.

Second, the field has become somewhat preoccupied with coding the contents of autobiographical memory and future thinking in terms of episodic and semantic content, and it is often assumed that more episodic detail represents better memory/future thinking [88]. However, we argue that such a perspective can be misleading. As we alluded to earlier, it is often difficult—if not impossible—to know for certain whether any particular detail that is reported in the context of a memory or future thinking protocol is episodic or semantic in nature. Given such uncertainty, it seems especially problematic to argue that more ‘episodic’ detail represents better memory/future thinking. For instance, a common finding from the ageing literature is that older, as compared with younger, adults report fewer ‘episodic’ and more non-episodic details when reporting on specific events (e.g. [45]). Such patterns of data fit neatly with extant ageing research demonstrating the difficulty that older adults tend to experience in accessing specific details from their past, leading to the interpretation that the increased generation of non-episodic details is to compensate for this loss [88,89]. However, this interpretation overlooks the fact that narrative content that contextualizes a specific event may support effective communication [50,90]. By moving away from a focus on the ‘episodic representation’ as traditionally conceptualized, we hope that our model will help to provide a more equal footing for studies of mental representations that, although more temporally general, conceptual or non-personal in nature, may nonetheless serve important functions in daily life.

Finally, our model raises the possibility that MTT and autonoesis may not be uniquely tied to episodic representations. There is good evidence that MTT is characteristic, albeit to differing degrees, for both specific and general events (e.g. [91]). Such findings imply that temporal specificity and to some extent representational content may not be critical for evoking a sense of reliving. Indeed, as we alluded to earlier, Conway [25] argues that ESK is not enough to evoke a sense of pastness, but that such details must be meaningfully connected to one’s self-concept. This suggests that MTT may be possible for more abstract representations of the personal past and future, such as lifetime periods. Anecdotally, such experiences are common in the context of revisiting familiar locations, such as taking a trip to an old neighbourhood; gathering with familiar people, such as in the context of reunions, weddings and funerals/celebrations of life; or simply being transported into a previous lifetime period by a familiar piece of music and feeling in the present what it felt like to be oneself in the past, often in the absence of any semblance of a specific memory. Indeed, the study of nostalgia is rife with examples of participants reporting feeling now what it felt like to be themselves in the past (e.g. [92]). Interestingly, most contemporary studies of nostalgia ask participants to report on specific episodes or events for which they may be nostalgic, but many participants report on lifetime periods (e.g. the good old days; [93]). Within the autobiographical memory literature, the close connection that has been theorized to exist between episodic memory and MTT often leads researchers to not measure MTT in relation to more abstract representations such as lifetime periods (e.g. [94]). When researchers do measure MTT in the context of lifetime periods, they tend to require participants to hone in on specific episodes associated with lifetime periods [95]. Investigating the sense of (p)re-living, or perhaps more appropriately (p)re-feeling, that accompanies more general aspects of the autobiographical past and future promises to provide important theoretical contributions to our understanding of MTT. Such studies would do well to assess the extent to which different aspects of the reported experiences relate to indices of MTT. For instance, it is possible that representations of lifetime periods might be accompanied by perceptual elements related to those periods and that ratings of MTT might map onto those elements. On the other hand, as D'Argembeau and others [e.g. 96] have shown, ratings of MTT might map more closely onto conceptual content, and so the extent to which a sense of MTT is associated with a lifetime period might depend, for instance, on the extent to which that lifetime period is considered central to one’s life story.

Our model is not without its limitations. We have already touched on general issues related to multidimensional models, including those regarding what dimensions to model and concerns around overfitting representational space. Moreover, it will be important for future work to determine whether, and if so how, to map real data to our model. For instance, will it be necessary to determine precisely where on the dimensions of temporal specificity, content and self-reference to situate mental representations of specific personal events, autobiographical facts and lifetime periods, or will it be satisfactory to use the emergent representational space to theorize and make predictions about the relation(s) among constructs of interest? This point may be moot given the transient nature of mental representations. The precise placement of a mental representation within our model is likely to depend on what it is about an event, fact or lifetime period that the individual happens to think about in the moment, how or whether the researcher measures different dimensions of the representation (e.g. behaviour, neuroimaging, etc.), and so on. Indeed, rather than attempt to directly map representations to models of multidimensional space, perhaps a more fruitful path forward would be a naturalistic one that uses such models to determine the extent to which specific dimensions of representation are relevant to everyday cognition. For instance, to what extent do individuals engage in thinking about specific as opposed to general, perceptual as opposed to conceptual, and idiosyncratic as opposed to shared aspects of experience? What factors determine the presence and/or absence of these dimensions in daily thought? What dimensions of representation provide critical links between mentation and behaviour? Whereas an emerging field of research on the facilitating effect of simulation on goal-directed action [97] suggests that episodic simulations that are specific, perceptual and idiosyncratic may support adaptive behaviour, it is also the case that more general and conceptual images of the self can serve to guide behaviour over extended periods of time [98]. We believe that more clearly delineating the dimensions of representation that guide behaviour in both the short and long term would represent a fruitful avenue for future research. Indeed, such questions could provide a better understanding of what dimensions of representation best capture the variance that underlies the complexities of cognition in daily life.

It will also be important to more fully develop how our model fits into developmental, comparative and neuropsychological considerations of the distinction between episodic and semantic memory. For instance, the developmental and comparative literatures have long been consumed by determining whether the many marvellous feats of behaviour that young children and non-human animals are able to accomplish might be explained by the capacity to represent the past and future in an episodic or ‘episodic-like’ fashion [99]. By turning attention away from an emphasis on episodicity as the holy grail of memory and future thinking, our model invites the possibility that it may not matter whether or not episodic cognition can explain the various behavioural feats of children and non-human animals, and that perhaps a more fruitful direction for future research will be to more precisely characterize the dimensions of representation that generally support such adaptive behaviours.

Further specification of our model from a neuroanatomical perspective will be necessary. Although we do not have the space to fully develop this perspective here, we have already highlighted inherent difficulties in making sense of neuroimaging data that appear to indicate that episodic and semantic systems are highly or even completely overlapping [54]. Similarly, even classic cases of amnesia that are often cited as evidence for an episodic/semantic distinction are not quite as clear-cut as they are made out to be in the literature. For instance, patient K.C. knew many things about their personal past that were not strictly episodic or semantic [100]. These realities are difficult to accommodate in the context of models that treat episodic and semantic memory as distinct, and often require the development of additional concepts to accommodate the data (e.g. personal semantics; [23]). Such realities are, however, more easily accommodated by models that focus instead on the multidimensional nature of mental representations, such that one does not need to resort to a fine distinction between categories that may nonetheless overlap across several underlying dimensions. Regardless, we acknowledge that more formal treatments of the dimensions that we outline and their relation(s) to various processing hierarchies of the brain will be valuable. At present, our model may best be thought of as a conceptual proposal that helps to better characterize the vast amount of mentation that can be represented and made available for task processing.

## 6. Conclusions

Forty years after Tulving’s [1] seminal book, the episodic–semantic distinction continues to exert a powerful influence on the scientific study of memory, and complex mental representations more broadly. However, as we have argued, this single axis makes it difficult to properly situate the various representations about the past, present and future that the human brain is capable of constructing and which can vary in terms of their temporal specificity, content and self-reference. Our Multidimensional Model of Mental Representations (MMMR) goes beyond the recent attempts to ‘rethink’ the distinction between episodic and semantic memory as overlapping yet distinct systems. We propose that these types of mentation are not at all distinct but rather are different outputs from one system that is fundamentally combinatorial and creates transient representations in the context of the ongoing stream of thought. Perhaps a more appropriate way to think about semantic and episodic representations is not as a distinction *per se* but rather as two (of many) goals of internal mentation that can be accomplished by a highly flexible constructive system.

## Data Availability

This article has no additional data.

## Footnotes

^1^
For clarity, we focus on explicit or declarative representations that, in addition to episodic and semantic memory, characterize a wide array of constructs that do not neatly fit the episodic–semantic distinction but that nonetheless emerge during mentation. Our review and model do not extend to implicit or non-declarative aspects of mental representation such as those that support classical conditioning, lower-level sensory processing, and other related phenomena.

^2^
Note that most theories and empirical research related to the episodic–semantic distinction have focused on memory. Nevertheless, MTT encompasses both past and future thinking, and based on the constructive episodic simulation hypothesis [8], we take the position that the issues related to the episodic–semantic distinction apply similarly to both past- and future-oriented cognition.

^3^
Other models deal with this issue by not including general semantic memory in the model at all (e.g. [20]).

^4^
This conflation of ‘episodic specificity’ at the level of content versus representation has added confusion to the literature [50–52]. Taken to an extreme, the exact combination of elements can be considered episodic or semantic depending on whether or not the representation is temporally specific and self-relevant.
